# Current Debates in the Management of Visceral Artery Aneurysms: Where the Guidelines Collide

**DOI:** 10.3390/jcm12093267

**Published:** 2023-05-04

**Authors:** Enrico Maria Marone, Luigi Federico Rinaldi

**Affiliations:** 1 Vascular Surgery, Department of Clinical-Surgical, Diagnostic and Pediatric Sciences, University of Pavia, Viale Brambilla 74, 27100 Pavia, Italy; 2 Department of Vascular Surgery, Policlinico di Monza Hospital, Via Amati 111, 20900 Monza, Italy; 3 Vascular Surgery, Department of Integrated Surgical and Diagnostic Sciences, University of Genoa, Viale Benedetto XV 6, 16132 Genoa, Italy

## 1. Current Debates in the Management of Visceral Artery Aneurysms: Where the Guidelines Collide

On one hand, the main difficulties in establishing a wide, evidence-based consensus about the best approach to visceral artery aneurysms (VAAs) and pseudoaneurysms (VAPAs) are the paucity of data, due to their rarity in the general population, and the extreme heterogeneity of this group of diseases, which encompasses different aneurysm types, with different degrees of rupture risks according to their anatomical locations [1].

These two aspects cause uncertainty and debate when it comes to defining treatment indications, especially in asymptomatic VAAs, and therapeutic strategies; this is mirrored by poorly evidenced and sometimes inconsistent statements in the current guidelines. Here, we review how the current European and American Guidelines address the most controversial issues regarding VAAs and VAPAs, namely the treatment of asymptomatic VAAs, the surveillance protocol in case of conservative treatment or after endovascular repair, the choice of treatment for patients with ruptured and unruptured aneurysms and pseudoaneurysms (endovascular vs. open), and the need for parent artery preservation or reconstruction.

### 1.1. Treatment Indications

The most recent European Society of Vascular and Endovascular Surgery (ESVS) Guidelines (last update in 2017) recommend the urgent treatment of patients with symptomatic VAAs and VAPAs (grade I, level C) and the elective treatment regardless of the size of asymptomatic VAPAs, VAAs of the pancreaticoduodenal (PDA) and gastric duodenal artery (GDA) aneurysms, intrahepatic hepatic artery (HA) aneurysms, and women of child-bearing age with VAAs and recipients of liver transplantation (grade IIb, level C) [2]. The surgical or endovascular treatment of patients with other asymptomatic VAAs is recommended only if the diameter is >25 mm (grade IIa, level C). Other, less recent national guidelines indicate different thresholds (e.g., 20 mm according to the Italian Society [3]).

The 2020 Society of Vascular Surgery (SVS) Guidelines agree on the indication to treat all ruptured (grade I, level A) and symptomatic VAAs and all VAPAs (grade I, level C), but they were the first to introduce distinctions based on locations regarding asymptomatic VAAs [4]. In fact, they agree with the European Guidelines on the treatment indication, irrespective of the size, of PDA and GDA aneurysms (grade I, level B), but they add to this group of aneurysms those of the gastric arteries (grade 1, level B), the superior mesenteric artery (SMA, grade I, level A), and the colic artery (grade 1, level B), whose rupture risk seems to be unrelated to the aneurysm sizes [1].

Other asymptomatic aneurysms should be surgically or endovascularly treated only if rapidly growing, or if their sizes exceed 2 cm for celiac trunk (CAAs), hepatic (HAAs), jejunal, and ileal aneurysms (grade I, levels B–C) and 3 cm for splenic artery aneurysms (SAAs, grade II, level C).

This distinction is based on the estimated rupture risk, which depends not only on the aneurysm size or growth rate, but also on its site and location, an aspect which is noted in many retrospective studies, although with a high degree of heterogeneity (notably, most recommendations are supported by a low or intermediate level of evidence). Moreover, unlike in the ESVS Guidelines, the indication for child-bearing aged women is expressed only for splenic artery aneurysms, whose ruptures during pregnancy with high fetal and maternal mortality are reported with a certain frequency [1].

### 1.2. Follow-Up Protocols

**Figure 1:**
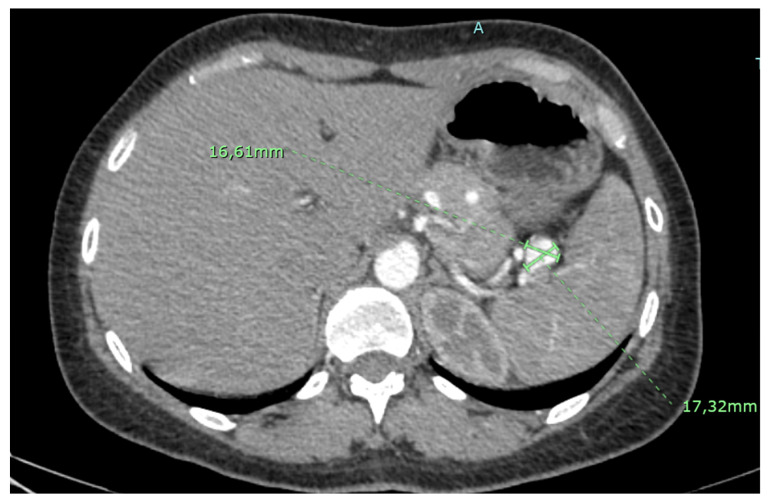
Small asymptomatic splenic artery aneurysm.

A conservative treatment with careful surveillance is recommended by both Guidelines for small asymptomatic aneurysms (Figure 1), with a discordance about the best follow-up protocol (every year according to the SVS, every 2–3 years according to the ESVS, with low evidence level in both cases).

**Figure 2:**
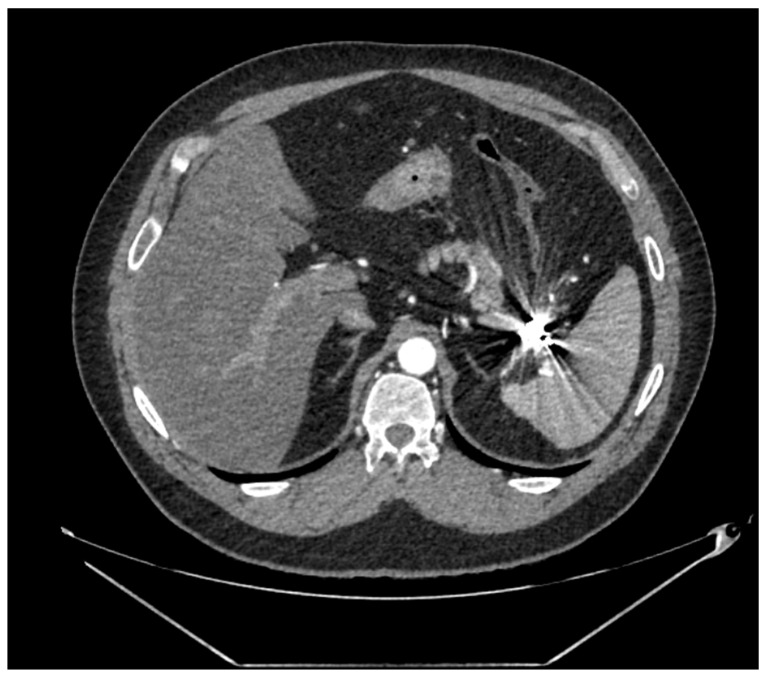
Post-operative CTA following coil embolization of a splenic artery aneurysm.

A post-operative follow-up does not necessitate imaging after an open repair, whereas endovascular therapy (EVT), Computed Tomography Angiography (CTA, Figure 2), or Magnetic Resonance Angiography is recommended every 24–48 months, depending on the aneurysm location, according to the SVS Guidelines (grade II, level B), and every 3 years according to the ESVS Document (grade IIb, level C).

**Figure 3:**
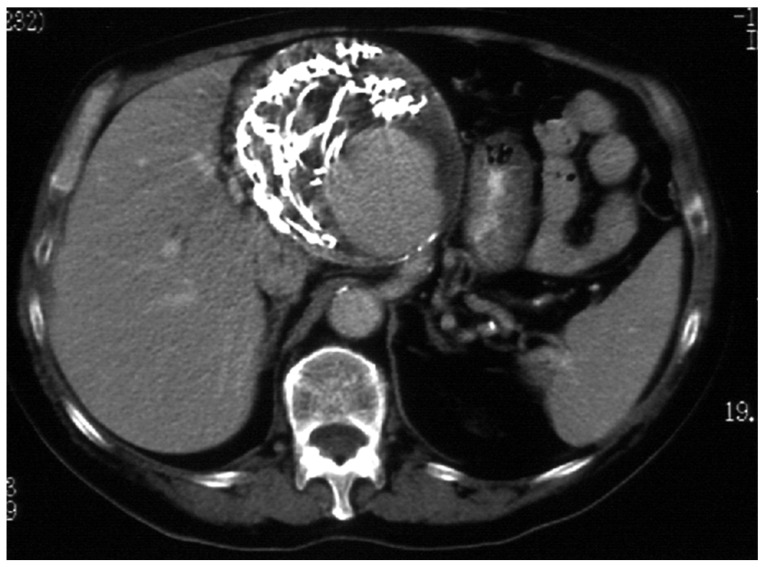
Post-operative CTA following common hepatic artery embolization.

**Figure 4:**
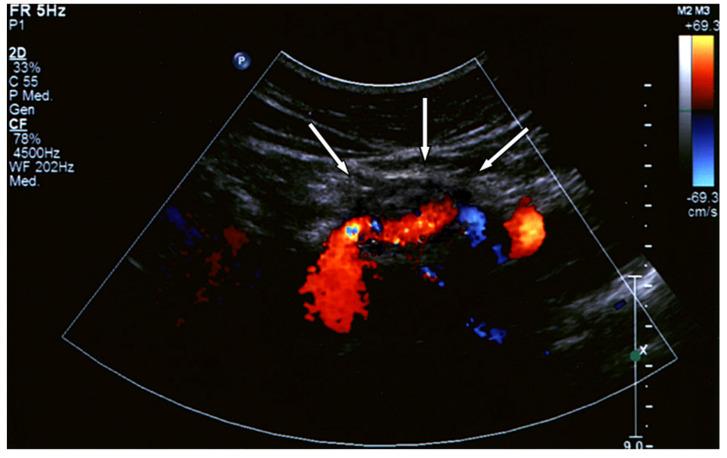
Post-operative CTA following covered stenting of a splenic artery aneurysm.

Distinctions made by the American Guidelines based on the aneurysm location generate more precise statements supported by higher levels of evidence [5]. However, the reliability of CTA following metallic coil embolization is still an unaddressed issue, which can produce imaging artifacts, making it difficult to evaluate optimal aneurysm exclusion or possible contrast extravasation, especially in case of small aneurysms (Figure 2 and Figure 3). The use of Color Doppler Ultrasound (Figure 4) as an alternative imaging tool in these cases is currently under investigation and is discussed in this Special Issue [6].

### 1.3. Treatment Choice

Despite the paucity of data, the two Guidelines share the view that endovascular treatment is burdened by lower perioperative mortality and morbidity rates, and so it should be preferred in anatomically suitable patients undergoing elective treatment. This recommendation is expressed as a general rule for all types of VAAs in the ESVS (grade IIa, level C) and for each visceral artery in the SVS Guidelines, albeit with different levels of evidence. However, the most updated meta-analysis conducted by Barrionuevo et al. reported a significantly lower peri-operative mortality for endovascular surgery only for PDAA/GDAA and coeliac artery aneurysms, whereas the general mortality rates are not significantly different between open and endovascular surgeries [1].

The therapeutic decision in the case of ruptured VAAs, especially in the case of hemodynamic instability, is even more controversial, mostly due to the rarity of studies and reports; moreover, most case series, systematic reviews, and meta-analysis do not distinguish their results based on ruptured/intact aneurysms, so peri-operative mortality and morbidity rates after the open vs. endovascular treatments of ruptured aneurysms are extremely difficult to estimate.

The presence of hemodynamic instability and the need to evacuate an intraperitoneal hematoma often require emergent laparotomy and open aneurysm resection, with artery ligation or reconstruction [5]. However, more people support the use of EVT as an emergent treatment for VAAs [7,8,9,10]. On the one hand, the ESVS Guidelines suggests that in this setting, “the benefits of endovascular treatment may be greater, as open repair for rupture is often more complex and results in a higher physiological insult”, without presenting a formal recommendation; on the other hand, the SVS Guidelines recommend both open or endovascular treatments of ruptured SAAAs and CAAs (levels IB and IA, respectively) and endovascular exclusion, mainly via embolization when more distal visceral arteries are involved (grades IB or IIB depending on the anatomic site). This distinction rely mainly on three considerations: the difficult surgical exposure and reconstruction of gastric, gastroduodenal, pancreatic duodenal arteries and intestinal branches of the mesenteric arteries, the demonstrated lower mortality rates after endovascular treatments (but limited to such anatomic locations), and the low risk of ischemia due to artery occlusion [1].

The difficulty of comparing the outcomes of open and endovascular treatments is further increased by the wide variety of endovascular techniques, from embolization to the use of covered of flow-diverting (FD) stents, and their different applications [11]. Among them, we must distinguish between those preserving the parent artery (covered stenting, flow diverters, and stent-assisted coiling) and those causing its occlusion (coil or liquid agent embolization).

**Figure 5:**
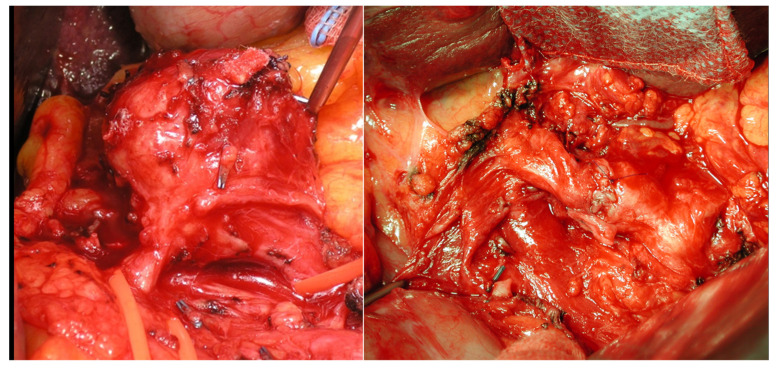
Open treatment of hepatic artery aneurysm with a direct suture reconstruction.

The choice must always consider not only anatomical suitability (i.e., proximal or distal localizations, the presence of adequate landing zones for stenting, etc.), but also the need to preserve the parent artery or the possible consequence of its intentional occlusion, and that is true in case of both EVT and open surgery (when choosing between artery ligation or reconstruction [12,13]. The ESVS Guidelines state that reconstruction should be preferred over occlusion techniques, when feasible, but we know from recent literature that embolization is widely used in emergent and elective settings, and most of the time, it is carried out without significant consequences. The SVS does not recommend routinary preservation of the parent artery in cases of SAAs, GAAs, GDAAs, PDAAs, and jejunal, ileal, and colic artery aneurysms; however, it does not mention if aneurysms could benefit of reconstruction or preservation in particular cases (e.g., proximal SSAs). Routine reconstruction/preservation is advised only in cases of extrahepatic HAAs (Figure 5), SMAAs, and CAAs if other mesenteric collaterals are diseased. Regarding specific techniques, only the SVS Guidelines provide precise indications, albeit limited to the cases in which parent artery preservation is not recommended, recommending coil embolization as the first choice and stent-assisted coiling or liquid agent embolization as the second choice.

Both Guidelines acknowledge the role of FDs as promising, but not yet fully elucidated, as well as the role of laparoscopic and robotic surgeries. The current literature provides the optimal results of these techniques, but the sample sizes are still too small to produce scientific evidence comparable to that of open or endovascular surgeries [14,15].

### 1.4. Debated Issues

In conclusion, based on the current scientific evidence, an integrated algorithm to guide decision making, including all treatment options for VAAs, cannot be constructed yet. Although the Guidelines agree on some basic aspects regarding the management of VAAs, many issues remain controversial, and they are based on scarce and low-quality data. Overall, the more recent SVS Document is more complete, but many questions are still unanswered and should be addressed by future investigations, including:

- The risk of the rupture of VAAs depending on their sizes, since the thresholds for elective treatment indicated by scientific societies are different;
- The real risk of the rupture of small VAAs of the SMA and its branches in order to ascertain if they really deserve interventional treatment regardless of their size;
- The best follow-up scheme for patients with intact VAAs at a low risk of rupture and the most suitable imaging technique for post-operative follow-up after endovascular treatment, especially coil embolization;
- The most appropriate diagnostic and therapeutic work-up for ruptured VAAs and the criteria for choosing between open and endovascular surgeries;
- The role of parent artery preservation in determining the decision between open and endovascular surgeries in both urgent and elective settings.

These aspects could be clarified only by collecting and analyzing more data and comparing different experiences in clinical settings. Therefore, all vascular specialists experienced in the field are welcome to contribute.
